# Finding measures of clinical placements quality for pre-service health services training: challenges of definition and search strategy construction

**DOI:** 10.1186/1472-6963-14-S2-P24

**Published:** 2014-07-07

**Authors:** Anne Cusick, Marie Heydon, Katherine Caldwell, Linda Cohen

**Affiliations:** 1 School of Health and Society, University of Wollongong, Wollongong, NSW, Australia; 2 Sydney Interdisciplinary Clinical Training Network, Health Workforce Australia, Rozelle, NSW, Australia; 3 Illawarra Health and Medical Research Institute, University of Wollongong, Wollongong, NSW, Australia; 4 Centre for Translational Neuroscience, University of Wollongong, Wollongong, NSW, Australia

## Background

Health services provide clinical training to medical, allied health and nursing staff. Ensuring the quality and quantity of this training is critical to workforce supply. Health Workforce Australia (HWA) has funded initiatives to enhance clinical training quality and the availability of clinical training placements. The quantity and quality of clinical placements must be increased. While there are many strategies to improve quantity and many strategies claim to enhance quality, there is an absence of agreed measures to evaluate placement quality. A standardised, comprehensive and widely applicable measure of clinical education placement quality is required. This study describes the challenges involved in constructing a systematic review search strategy.

## Materials and methods

An HWA document audit was conducted to examine the use of the term “quality”, identify attributes and locate any standardised measure of placement quality recommended. As none was found, a search strategy for a systematic review of literature was developed.

## Results

Quality clinical placements have not yet been conceptually or operationally defined by HWA. “clinical placements” were defined by HWA in 2012 using the qualifying term of “pre-service” or “pre-registration”. Their definition of quality clinical placements links student learning outcomes with high quality clinical learning environments. While characteristics of quality clinical learning environments, the student experience and learning outcomes have been proposed in literature, to date a standard definition has not been adopted.

Precise search terms are required to locate published information relating to measuring quality clinical placements and instruments that might measure them. The absence of a standard definition thus creates a challenge. Unsurprisingly previous literature reviews on quality clinical placements had not used standardized search terms.

Careful inspection of thesauri and search engines revealed a complexity and inconsistency of terminology surrounding clinical placement quality that was both unexpected and confusing. There was no term that matched ‘quality clinical placements’ and a vast array of terms had to be inspected, definitions interrogated and selections made.

MeSH terms provide the greatest precision and scope, however they were not used in CINAHL or Google Scholar. Equivalent terms had to be identified. Search engines that were most productive were: PubMed, CINAHL and Google Scholar. There was overlap in many CINAHL and PubMed sources; Google Scholar revealed highly relevant sources such as commissioned reports and position papers that were not detected through PubMed or CINAHL searches. No standardised measure for evaluation of clinical placement quality was identified.

